# Quantification of left ventricular mass by echocardiography compared to cardiac magnet resonance imaging in hemodialysis patients

**DOI:** 10.1186/s12947-020-00217-y

**Published:** 2020-09-16

**Authors:** Sören Jendrik Grebe, Uwe Malzahn, Julian Donhauser, Dan Liu, Christoph Wanner, Vera Krane, Fabian Hammer

**Affiliations:** 1 Department of Medicine I, University Hospital of Würzburg, Würzburg, Germany; 2 Department of Paediatrics, University Hospital of Erlangen, Loschgestraße 15, 91054 Erlangen, Germany; 3 Clinical Trial Center Würzburg, University Hospital Würzburg, Würzburg, Germany; 4 Department of Diagnostic and Interventional Radiology, University Hospital of Würzburg, Würzburg, Germany; 5 Division of Cardiology, University of Würzburg, Würzburg, Germany; 6 Comprehensive Heart Failure Centre, University Hospital and University of Würzburg, Würzburg, Germany; 7 Department of Internal Medicine B, Division of Cardiology, University Medicine, Greifswald, Germany

**Keywords:** Teichholz formula, ASE formula, Echocardiography, Left ventricular hypertrophy, Left ventricular mass index, Hemodialysis

## Abstract

**Background:**

Left ventricular hypertrophy (LVH), defined by the left ventricular mass index (LVMI), is highly prevalent in hemodialysis patients and a strong independent predictor of cardiovascular events. Compared to cardiac magnetic resonance imaging (CMR), echocardiography tends to overestimate the LVMI. Here, we evaluate the diagnostic performance of transthoracic echocardiography (TTE) compared to CMR regarding the assessment of LVMI in hemodialysis patients.

**Methods:**

TTR and CMR data for 95 hemodialysis patients who participated in the MiREnDa trial were analyzed. The LVMI was calculated by two-dimensional (2D) TTE-guided M-mode measurements employing the American Society of Echocardiography (ASE) and Teichholz (Th) formulas, which were compared to the reference method, CMR.

**Results:**

LVH was present in 44% of patients based on LVMI measured by CMR. LVMI measured by echocardiography correlated moderately with CMR, ASE: r = 0.44 (0.34–0.62); Th: r = 0.44 (0.32–0.62). Compared to CMR, both echocardiographic formulas overestimated LVMI (mean ∆LVMI (ASE-CMR): 19.5 ± 19.48 g/m^2^, *p* < 0.001; mean ∆LVMI (Th-CMR): 15.9 ± 15.89 g/m^2^, *p* < 0.001). We found greater LVMI overestimation in patients with LVH using the ASE formula compared to the Th formula. Stratification of patients into CMR LVMI quartiles showed a continuous decrease in ∆LVMI with increasing CMR LVMI quartiles for the Th formula (*p* < 0.001) but not for the ASE formula (*p* = 0.772). Bland-Altman analysis showed that the Th formula had a constant bias independent of LVMI. Both methods had good discrimination ability for the detection of LVH (ROC-AUC: 0.819 (0.737–0.901) and 0.808 (0.723–0.892) for Th and ASE, respectively).

**Conclusions:**

The ASE and Th formulas overestimate LVMI in hemodialysis patients. However, the overestimation is less with the Th formula, particularly with increasing LVMI. The results suggest that the Th formula should be preferred for measurement of LVMI in chronic hemodialysis patients.

**Trial registration:**

The data was derived from the following clinical trial: NCT01691053, registered on 19 September 2012 before enrollment of the first participant.

## Background

Left ventricular hypertrophy (LVH) is highly prevalent in patients on hemodialysis [1–4]. LVH, as measured by the left ventricular mass index (LVMI), is a strong predictor of cardiovascular disease (CVD) [3, 4]. Consequently, a reliable and valid method to detect LVH is needed for both clinical and scientific implications [5, 6]. For the assessment of left ventricular mass (LVM), cardiac magnetic resonance imaging (CMR) has been established as the most accurate and reproducible method [7–9]. However, given its limited availability and high cost, CMR is not practical for clinical use in large-scale clinical studies. In contrast, the two-dimensional (2D) targeted M-mode echocardiography is preferred in the clinical context because of its widespread availability, low cost, simple handling, and extensive evidence base [10]. Nevertheless, echocardiographic linear measurement and LVM calculation by cube function formulas have their own limitations [10–13]. The current recommended formula from the American Society of Echocardiography (ASE) is based on special geometric assumptions, which may become inaccurate in the presence of asymmetric hypertrophy, eccentric remodeling, or distortion of left ventricular (LV) geometry and may lead to an incremental overestimation of LVMI [3, 14–17]. Teichholz et al. designed a formula that includes a volume-correcting function in order to minimize the error inter alia in patients with LVH [18]. A recent CMR study investigating patients with aortic stenosis demonstrated that the Teichholz (Th) formula had a lower tendency to overestimate the value within a population with increased LVMI [17].

Here, we investigated the performance of two echocardiographic formulas, ASE and Th, in calculating LVMI in patients on hemodialysis.

## Methods

### Study

All echocardiography and CMR data were acquired in the context of the Mineralocorticoid Receptor Antagonists in End-Stage Renal Disease (MiREnDa), which was a prospective, randomized, placebo-controlled, double-blind, parallel group, phase 2 intervention study investigating the efficacy and safety of spironolactone in hemodialysis patients. The study design, flow, and endpoints were reported previously [19, 20]. Briefly, patients were enrolled at 20 dialysis centers. Cardiac imaging was assessed in one of the three participating university centers (Frankfurt, Erlangen-Nuremberg, and Würzburg) according to predefined standardized procedures. The patients underwent three dialysis sessions per week. To avoid inaccuracies in measuring LVM, CMR and echocardiography were performed immediately after each other for all subjects on dialysis-free days. Data from CMR and echocardiography were collected after the placebo run-in phase and before randomization (week 0 visit). The coordinating university for the study was the University Hospital of Würzburg. The Center for Clinical Trials at the University Hospital of Würzburg was responsible for implementing the study and the statistical analyses. Blinded data assessment was performed by trained research staff [19, 20]. The data used in this analysis were collected before the double-blind treatment phase.

### Echocardiography

All patients underwent a comprehensive echocardiographic study by an experienced cardiologist blinded to CMR findings. The protocol based on the current ASE recommendations utilized linear measurements derived by 2D TTE-guided M-mode approach [5, 13]. LVM was calculated from the TTE measurements using the following equations:

ASE formula [5, 21]

$$ =1.04\times \left\{{\left( LVEDd+ PWTd+ IVSTd\right)}^3-{LVEDd}^3\right\}\times 0.8+0.6 $$

Th formula [18]

$$ =1.04\times \left\{\left[\raisebox{1ex}{$7.0$}\!\left/ \!\raisebox{-1ex}{$\left(2.4+ LVEDd+ PWTd+ IVSTd\right)$}\right.\right]\times {\left( LVEDd+ PWTd+ IVSTd\right)}^3-\left[\raisebox{1ex}{$7.0$}\!\left/ \!\raisebox{-1ex}{$\left(2.4,+, LVEDd\right)$}\right.\right]\times {LVEDd}^3\right\} $$

Where LVIDd is the LV internal diameter at end-diastole, PWTd is the posterior wall thickness at end-diastole, and IVSTd is the interventricular septal thickness at end-diastole. LVM indexation to anthropometry was calculated by body surface area (BSA) using the Mosteller formula [22]. In addition, the indexation by height^2.7^ was used [23]. In this case, the LVMI is marked accordingly as LVM/height^2.7^.

### CMR imaging

CMR images were acquired using both 1.5 or 3 Tesla magnetic resonance imaging scanners. Imaging was performed using an electrocardiogram-gated, breath-hold, 2D, steady-state, free precession cine with contiguous, left ventricular short-axis stack of images acquired from above the base to below the apex of the left ventricle with a slice thickness of 8.0 mm, pixel size smaller than 1.4 mm × 1.4 mm, field of view ~ 320 mm (adapted to patient size), and temporal resolution < 50 ms.

Image analysis was performed using commercial software (Circle cvi42®). LV myocardial area slices were measured planimetrically by delineating endocardial and epicardial LV borders at end-diastole and end-systole, allowing calculation of end-diastolic and end-systolic volumes, as well as myocardial volumes. LVM was calculated by summing the short axis slice volumes at end-diastole. Papillary muscles and trabeculae mass (PMT) were included in the LV cavity volume. LVM was mainly indexed to BSA using the Mosteller formula (see above). LVH was defined as indexed LVM ± 2 standard deviations of CMR references [24].

Scans were analyzed by an experienced radiologist (J.D.) blinded to the results of echocardiography. To validate CMR data, a second independent radiologist analyzed a random subsample of 26 (13%) CMR scans according to the same predefined procedures [20].

### Statistical analysis

Mahalanobis distance was used to detect outliers in the data set and data from one subject was excluded from analysis due to its strong statistical divergence from the other LVMI measurements in population. A further exclusion criterion was the absence of data from CMR or echocardiography. Continuous data were expressed as median (quartiles) and categorical data as frequencies (percentages). If data matched the assumption of normal distribution (Shapiro-Wilk test, *p* > 0.05), continuous data in independent groups in paired samples were compared by Student’s unpaired or paired *t* test (k = 2) or single factor variance analysis (k > 2 independent groups), otherwise Mann-Whitney U/Wilcoxon sign rank test (k = 2) or the Kruskal Wallis test (k > 2) was used. Distributions for categorical data were compared using χ^2^ or Fisher’s exact test if appropriate. The degree of correlation and its confidence interval were computed using Concordance Correlation Coefficient [25]. The mean difference in LVMI between CMR and echocardiography was calculated according to the formula $\overline{\varDelta}$ = $\frac{1}{n}\sum \limits_{i=1}^n{\left( Echo- CMR\right)}_i$. Agreement between the CMR technique and echocardiographic methods was analyzed by the Bland-Altman plot. The bias was estimated by the mean difference between CMR and echocardiography. In the case of constant bias, evaluated by the regression line, we specified the 95% limits of agreement. The stratification into LVMI quartiles was based on CMR measurements of LVMI. To compare the diagnostic performance of both echocardiographic formulas, we performed receiver operating characteristics (ROCs) to calculate the area under the curve (AUC). To test its significance, we used the method by Hanley et al. If z ≥ 1.96, the null hypothesis AUC_1_ = AUC_2_ was rejected by means of the z-test with a significance level of 0.05 and the so-called true ROC areas taken as significantly different [26]. Cut-off values for classifying LVH status were calculated by the Youden index. A two-tailed *p-*value < 0.05 was considered significant. For this purely exploratory analysis, no corrections for multiple comparisons were made. Statistical analyses were conducted using SPSS Version 19.0 (IBM Corp., Armonk, NY, USA) and Excel 2016 (Microsoft Corp., Redmond, USA).

## Results

### Demographics

A total of 118 hemodialysis patients were enrolled in the MiREnDa trial. Valid data sets for CMR and echocardiographic were available for 95 patients (mean age: 60 (50.0–71.0) years; 76% males) with a mean body mass index of 26.3 kg/m^2^. Patients had been on maintenance hemodialysis for a median duration of 42 (16.8–79.0) months, which was performed via an arteriovenous fistula in 93% of patients. Based on CMR measurements, LVH was present in 44% of patients (69% concentric and 31% eccentric hypertrophy). There was a high prevalence of hypertension (86%), whereas heart failure was infrequent (5%). Medical treatment included beta-blockers (67.4%), diuretics (54.7%), and angiotensin-converting enzyme inhibitor or angiotensin-receptor blocker (52.6%). We found no significant differences between patients with and without LVH except for renin-angiotensin-system (RAS) blockade and ß-blocker intake (Table 1).

**Table 1:** Baseline characteristics

	All patients (*n* = 95)	LVH - (*n* = 53)	LVH + (*n* = 42)	*P*-value
Age, years	60.0 (50.0–71.0)	59.0 (49.5–68.0)	64 (51.0–72.0)	0.258
Males, n (%)	72 (75.8%)	43 (81.1%)	29 (69.0%)	0.172
Height, m	1.72 (1.66–1.80)	1.72 (1.66–1.81)	1.72 (1,66–1.79)	0.642
Weight, kg	79.0 (68.5–95.0)	86.0 (66.0–102.0)	74.6 (69.0–88.0)	0.066
BMI, kg/m^2^	26.3 (23.9–31.2)	27.9 (23.8–32.4)	25.4 (23.9–28.5)	0.119
BSA, m^2^	1.97 (1.79–2.16)	2.02 (1.77–2.22)	1.90 (1.79–2.10)	0.121
Height^2.7^	4.3 (3.9-4.9)	4.3 (3.9-4.9)	4.3 (3.9-4.8)	0.756
Co-morbidities, n (%)				
Hypertension	86 (90.5)	48 (90.6)	38 (90.5)	1.000
CV events	15 (15.8)	8 (15.1)	7 (16.7)	0.835
CHD	33 (34.7)	19 (35.8)	14 (33.3)	0.798
Heart failure	5 (5.3)	3 (5.7)	2 (4.8)	1.000
Diabetes mellitus	28 (29.5)	13 (24.5)	15 (35.7)	0.235
Medication, n (%)				
Beta-blocker	64 (67.4)	30 (56.6)	34 (81.0)	0.012
RAS blockade	52 (54.7)	24 (45.3)	28 (66.7)	0.038
Diuretics	50 (52.6)	24 (45.3)	26 (61.9)	0.107
CCB	44 (46.3)	21 (39.6)	23 (54.8)	0.142
Statins	29 (30.5)	16 (30.2)	13 (31.0)	0.936
Dialysis characteristics				
Dialysis duration, months	42.5 (16.8–79.0)	45.0 (17.0–69.0)	42.0 (16.0–85.0)	0.750
Fistula, n (%)	88 (92.6)	48 (90.6)	40 (95.2)	0.148

Data are presented as median (quartiles) or prevalence (percent). *LVH* left ventricular hypertrophy; *BMI* body mass index; *BSA* body surface area (Mosteller formula); *CV* cardiovascular; *CHD* coronary heart disease; *RAS* renin-angiotensin-system; *CCB* calcium channel blockers

### Assessment of LVM/BSA differences by echocardiographic methods and CMR

**Fig. 1:**
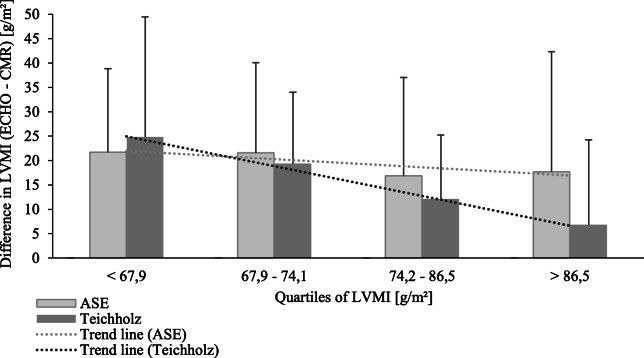
Mean difference in LVMI derived from CMR and echocardiographic formulas stratified by LVMI quartiles. The dark and light gray dotted lines depict the trendlines for mean differences in Teichholz (right) and ASE (left) formulas, respectively, across LVMI quartiles. Data are presented as mean +  standard deviation (SD). CMR, cardiac magnetic resonance; LVMI, left ventricular mass index; ASE, American Society of Echocardiography

Echocardiographic and CMR parameters, including LVM and LVMI, are provided in Table 2. LVMI measured by echocardiography was significantly higher than that measured by CMR (*p* < 0.001 for both ASE and Th). LVMI according to CMR correlated only moderately with LVMI derived from both echocardiographic formulas (Table 3). Furthermore, both echocardiographic formulas significantly overestimated LVMI compared to CMR. The degree of LVMI overestimation by ASE was significantly greater than that of Th in the entire cohort and in patients with LVH, but not in those without LVH (Table 4). Compared to the $\overline{\varDelta}$ LVMI between echocardiographic formulas and CMR according to LVMI quartiles, we found a significantly lower difference in higher (3rd and 4th) LVMI quartiles for the Th formula compared to the ASE formula, whereas the ASE formula indicated a significantly smaller mean difference in the first LVMI quartile. With regard to the change in $\overline{\varDelta}$ LVMI according to LVMI quartiles, only the Th formula revealed diminishing $\overline{\varDelta}$ LVMI with increasing LVMI quartile (1st quartile, 24.8 ± 15.0 g/m^2^; 2nd quartile, 19.4 ± 14.7 g/m^2^; 3rd quartile, 12.1 ± 13.1 g/m^2^; 4th quartile, 6.8 ± 17.4 g/m^2^; *p* < 0.001), whereas the ASE formula resulted in no significant difference between the quartile groups, suggesting a better performance of the Th formula with higher LVMI (Table 4, Fig. 1).

**Table 2:** Echocardiographic and cardiac magnetic resonance characteristics

	All patients	LVH –	LVH +	*P*-value
CMR parameters				
EDV, ml	158.7 (126.1–190.4)	138.4 (113.0–160.1)	184.5 (160.8–217.1)	< 0.001
LVM, g	146.6 (122.7–176.4)	134.1 (108.2–148.7)	158.7 (151.3–200.8)	< 0.001
LVMI, g/m^2^	74.2 (62.3–86.4)	66.9 (59.0–73.6)	87.5 (81.3–100.4)	< 0.001
LVMI, g/m^2.7^	33.8 (29,3-39,2)	30.6 (25.4-32.8)	40.8 (36.9-44.4)	< 0.001
Echocardiographic parameters				
LVIDd, mm	46.0 (43.0–52.0)	45.0 (41.5–49.0)	48.0 (44–53.3)	0.006
PWTd, mm	11.0 (10.0–11.0)	10.0 (9.0–11.0)	11.0 (10.0–12.0)	< 0.001
IVSTd, mm	11.0 (10.0–12.0)	10.0 (10.0–11.5)	12.0 (11.0–12.0)	< 0.001
LVM (ASE), g	181.2 (153.2–213.9)	164.5 (135.4–197.3)	206.7 (174.7–237.3)	< 0.001
LVMI (ASE), g/m^2^	94.4 (77.3–108.3)	82.6 (66.6–99.9)	104.2 (92.6–122.5)	< 0.001
LVMI (ASE), g/m^2.7^	42.3 (35.9–50.3)	37.8 (30.4-45.1)	48.9 (42.1-59.7)	< 0.001
LVM (Th), g	178.7 (156.7–195.2)	165.4 (146.9–184.8)	186.3 (174.8–219.0)	< 0.001
LVMI (Th), g/m^2^	91.7 (78.6–101.8)	83.0 (71.4–94.3)	100.9 (89.9.112.2)	< 0.001
LVMI (Th), g/m^2.7^	42.1 (35.8–46.6)	38.3 (32.3-42.5)	45.7 (42.0-52.7)	< 0.001

Data are presented as median (quartiles); *LVH* left ventricular hypertrophy; *CMR* cardiac magnetic resonance; *EDV* end diastolic volume; *LVM* left ventricular mass; *LVMI* left ventricular mass index; *LVIDd* LV internal diameter at end-diastole; *PWTd*, the posterior wall thickness at end-diastole; *IVSTd*, interventricular septal thickness at end-diastole; *ASE* Anerican Society of Echocardiography; *Th* Teichholz

**Table 3:** Concordance Correlation Coefficients between LVMI calculated by echocardiography formulas (ASE, Teichholz) and cardiac magnetic resonance imaging

	ASE	Teichholz
All patients	0.44 (0.34–0.62)	0.44 (0.32–0.62)

Concordance Correlation Coefficient (confidence interval). *ASE* American Society of Echocardiography; *LVH* left ventricular hypertrophy

**Table 4:** Mean differences between LVMIs calculated by ASE and Teichholz formulas and cardiac magnetic resonance imaging stratified by presence/absence of left ventricular hypertrophy and by quartiles of LVMI

	ΔLVMI, g/m^2^ (ASE-CMR)	ΔLVMI, g/m^2^ (Teichholz-CMR)	*P-*value
All patients (n = 95)	19.5 ± 20.1***	15.9 ± 16.4***	0.001
LVH – vs. LVH +			
LVH – (*n* = 53)	18.6 ± 18.0***	18.5 ± 15.0***	0.93
LVH + (*n* = 42)	20.6 ± 22.6***	12.6 ± 17.5***	< 0.001
LVH – vs. LVH +	***p*** **= 0**.**645**	***p*** **= 0.077**	
LVMI quartiles			
LVMI Q1 (< 67.9 g/m^2^)	21.7 ± 17.1***	24.8 ± 15.0***	0.030
LVMI Q2 (67.9–74.1 g/m^2^)	21.6 ± 18.5***	19.4 ± 14.7***	0.146
LVMI Q3 (74.2–86.5 g/m^2^)	16.9 ± 20.2***	12.1 ± 13.1***	< 0.050
LVMI Q4 (> 86.5 g/m^2^)	17.7 ± 24.7**	6.8 ± 17.4	< 0.001
Overall comparison of quartiles	***p*** **= 0.772**	***p*** **< 0.001**	

Data are presented as mean ± standard deviation; *LVMI* left ventricular mass index; *ASE* American Society of Echocardiography; *Th* Teichholz.; *Q* quartile; *LVH* left vetricular hypertrophy. The *P* value between both echocardiographic formulas is depicted in the right-hand column; a significant difference between echocardiographic and CMR measurements was indicated by *** *P* < 0.001, ** *P* < 0.01, and * *P* < 0.05; the overall comparison of quartiles was performed by single factor variance analysis.

**Fig. 2:**
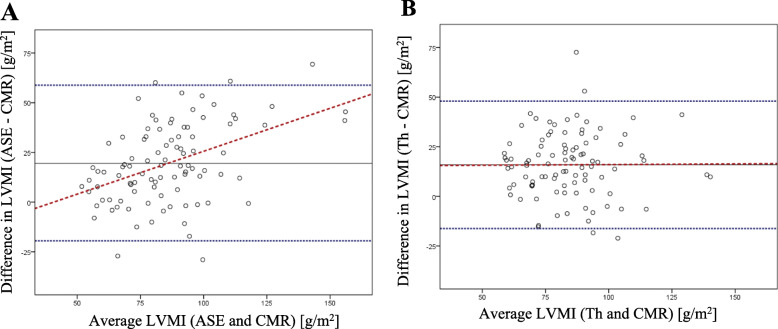
Bland-Altman plots. LVMI based on echocardiography was calculated by the (**a**) ASE formula or the (**b**) Teichholz formula. The black line depicts the average difference between each echocardiography and CMR measurement, the dotted blue lines depict the 95% limits of agreement (average difference ± 1.96 × standard deviation), and the red dotted line describes the regression line

The agreement between LVMI derived from echocardiographic methods and CMR was further evaluated by the Bland-Altman analysis. The differences between LVMI were plotted against the average LVMI, which showed poor agreement between LVMI calculated by both echocardiographic formulas and the LVMI measured by CMR. Both echocardiographic formulas demonstrated systematic overestimation. However, in contrast to ASE, the Th formula had a constant bias that did not vary throughout the range of measurements. The 95% limits of agreement were 19.7 to 46.2 g/m^2^. Conversely, the $\overline{\varDelta}$ LVMI (ASE-CMR) had an ascending regression line with increasing LVMI, suggesting that the bias was related to the LVMI (Fig. 2).

### ROC-AUC analysis

The ROC-AUC analysis of diagnostic performance was good for both formulas. The AUC for Th was not statistically better than ASE (Th: 0.819 (0.737–0.901) vs. ASE: 0.808 (0.723–0.892), z = 0.039) [26]. The calculations for LVM/height^2.7^ showed similar results (Th: 0.814 (0.730–0.898) vs. ASE: 0.801 (0.730–0.898), z = 0.047). The non-sex-specific cut-off value for the Th formula calculated by the Youden index was 83.5 g/m^2^ and 42.1 g/m^2.7^. Using this cut-off values, the prevalence of LVH was 68.4 and 49.5% respectively.

## Discussion

This study assessed the diagnostic accuracy of echocardiography in determining the LVMI in hemodialysis patients. The two echocardiographic formulas correlated moderately with CMR and overestimated LVMI compared to CMR measurements. The overestimation of LVM using the Th formula was less severe in patients with higher LVMI values. Therefore, these analyses showed an advantage of the Th formula in calculating LVMI in chronic hemodialysis patients with LVH but not in the absence of LVH.

This phenomenon might be explained by the intrinsic difference between the formulas for estimating the left ventricular volume (LVV). This becomes evident once the true LV geometry differs from the assumed ellipsoidal geometry. Though CMR imaging or 3D echocardiography measures myocardial volume to quantify LVM, 2D targeted linear m-mode quantification by echocardiography relies on geometric assumptions considering a left ventricle shape approximately equivalent to a prolate ellipsoid (LVV = ¾πr_1_r_2_r_3_) [27]. The basic assumption of the cube function is that the minor radii (r_1_ and r_2_) of the prolate ellipsoid are half the major radius (r_3_) [28]. In patients with increasing LVM, there is probably an incremental change in the configuration as a result of an increasing short axis accompanied by a gradual decrease in the long axis [18]. Prior studies using the ASE formula have also shown that measurement of LVMI by 2D targeted M-mode echocardiography results in overestimation of LVMI [15, 29–33]. Thus, the ASE formula is recommended for use in patients without major distortions in LV geometry [5]. The approach developed by Teichholz et al. for correcting systematic errors in the LVV formula attempts to take changes of configuration into account and correct the cube function formula to LVV = [7.0/2.4 + r](D)^3^ as an adaption to the observed curvilinear changes in the enlarging left ventricle [17, 18]. This component of the Th formula may also be the reason for the declining trend in ΔLVMI described above.

Notably, the original study by Teichholz et al. analyzed the relationship between the long and short axes using an out-of-use angiographic technique. However, a recent CMR study confirmed these findings, demonstrating that the left ventricle tends to become more spherical with increasing LVM [17]. This study enrolled 99 Asian patients with aortic stenosis and compared the LVMI calculated by the ASE and Th formulas. The authors demonstrated a significant overestimation of both echocardiographic formulas compared to CMR. However, a comparison of the two echocardiographic formulas revealed a significantly lower overestimation of LVMI by the Th formula in patients with LVH compared to those without, which is in good agreement with our findings in hemodialysis patients.

In contrast to these findings, a necropsy validation study by Devereux et al. demonstrated a systematic underestimation of LVM by the Th formula, which was particularly obvious in patients with LVH [21]. Interim methodological and technological changes may explain this inconsistency. First of all, animal necropsy validation studies of CMR seem to indicate that assessing LVM by manual planimetry underestimates LVM [33]. Secondly, the echocardiography ultrasound frequency has changed, and fundamental imaging (FI) was replaced by harmonic imaging (HI) to improve the definition of pericardial and epicardial borders [6, 21]. This FI technique has been shown to underestimate LVM values compared to CMR, whereas the HI technique, which we used, seemed to provide higher LVM values [34]. Another point to take into consideration is the exclusion of the PMT of the LV by CMR. Studies have demonstrated that PMT contributes 6.2 to 15.5% [35] of the total LVM and significantly affects LVM quantification [35, 36]. For example, Janik et al. compared one-dimensional LVM based on the ASE formula to the three-dimensional method derived by CMR using manual planimetry. The inclusion of PMT yielded significantly lower mean LVM differences between these methods, and the difference was 3-fold higher among patients with concentric and eccentric hypertrophy [35]. All of these aspects could make an additional contribution to the discrepancy of CMR and TTE measurement of LVM, which cannot necessarily be explained by methodological differences alone [33].

LVM is traditionally indexed by body surface area (BSA) [22] but other indexations have also been used [6]. In dialysis patients, indexation by height^2.7^ has been shown to be somewhat superior to BSA with regard to predicting cardiovascular mortality [3]. However, several studies showed that indexation by BSA has a similarly high prognostic value and is still recommended by ASE [13]. Given that the focus of this study was on the comparison of two methods we chose indexation by BSA which also allowed us to compare our findings with previous studies.

Our study is the first to compare the determination of LVMI by echocardiography using the Th and ASE formulas to CMR findings in hemodialysis patients. The strengths of this analysis are that the echocardiographic and CMR studies were performed according to standard operating procedures defined before the start of the MiREnDa study [20].

The limitations of our study are the limited sample size and low percentage of woman. We acknowledge the lack of patients with higher LVMI for consistent evaluation of the trend of overestimation towards higher LVMI, and the limitations of our cut-off value for the Th formula. Geometric changes in patients with LVH measured by CMR could not be analyzed, as it was not part of the MiREnDa CMR protocol. In addition, the LVM measured by CMR was calculated by exclusion of PMT, although there are recent data for normal values with inclusion of PMT [37]. A further limitation is the lack of any reproducibility analysis. Furthermore, our findings likely cannot be extrapolated to patients receiving peritoneal dialysis or to non-white hemodialysis patients.

## Conclusion

In conclusion, this study demonstrates that the Th formula outperforms the ASE formula in calculating the LVMI in hemodialysis patients, particularly in those with LVH. Further studies are necessary to test the Th formula in larger cohorts of hemodialysis patients with higher LVM and to address and compare the prognostic value of LVM derived from the Th formula compared to the ASE formula.

## Data Availability

The datasets generated and analyzed during the current study are not publicly available due to data privacy reasons but are available from the corresponding author on request.

## Abbreviations

2D: Two-dimensional
ASE: American Society of Echocardiography
AUC: Area under the curve
BSA: Body surface area
CMR: Cardiac magnetic resonance
EDV: End diastolic volume
FI: Fundamental imaging
HI: Harmonic imaging
IVSTd: Interventricular septal thickness at end-diastole
LV: Left ventricular
LVH: Left ventricular hypertrophy
LVIDd: LV internal diameter at end-diastole
LVM: Left ventricular mass
LVMI: Left ventricular mass index
LVV: Left ventricle volume
MiREnDa: Mineralocorticoid Receptor Antagonists in End-Stage Renal Disease
PMT: Papillary muscles and trabeculae mass
PWTd: The posterior wall thickness at end-diastole
RAS: Renin-angiotensin-system
ROC: Receiver operating characteristic
SD: Standard deviation
Th: Teichholz
TTE: Transthoracic echocardiography
